# Correlates and Longitudinal Renal and Cardiovascular Implications of FGF23 Levels in HIV-Positive Individuals

**DOI:** 10.1371/journal.pone.0155312

**Published:** 2016-05-13

**Authors:** Mohamed G. Atta, Michelle M. Estrella, Derek M. Fine, Katie Zook, Jose Manuel Monroy Trujillo, James H. Stein, Gregory M. Lucas

**Affiliations:** 1 Johns Hopkins University School of Medicine, Department of Medicine, Baltimore, MD, 21287, United States of America; 2 Department of Medicine, Cardiovascular Medicine Division, University of Wisconsin School of Medicine and Public Health, Madison, Wisconsin, 53792, United States of America; Azienda ospedaliero-universitaria di Perugia, ITALY

## Abstract

Fibroblast growth factor23 (FGF23), an early marker of kidney dysfunction, is associated with cardiovascular death. Its role in HIV-positive individuals is unknown. We measured FGF23 in 100 HIV-negative and 191 HIV-positive nondiabetic adults with normal baseline estimated glomerular filtration rate (GFR). We measured GFR by iohexol annually, albumin-creatinine ratio (ACR) every 6 months, as well as pulse wave velocity, carotid plaque, and carotid intima media thickness (IMT) at baseline and 2 years. Progressive albuminuria was defined as follow-up ACR ≥2-fold than baseline and ≥30 mg/g. Regression models assessed associations of FGF23 with baseline factors and longitudinal changes in disease markers. FGF23 levels were similar in HIV serostatus. Among HIV-positive persons, factors independently associated with higher baseline FGF23 levels included female (adjusted ratio of geometric means [95% CI],1.46 [1.21,1.76]), serum phosphorus (1.20 [1.03,1.40]), HCV (1.31 [1.10,1.56]) and non-suppressed HIV RNA (1.27 [1.01,1.76]). At baseline, FGF23 was not associated with GFR, albuminuria, carotid plaque, or carotid IMT in cross-sectionally adjusted analysis of HIV-positive individuals. However, higher baseline FGF23 was associated with progressive albuminuria (odds ratio1.48 [95% CI]:1.05,2.08) and a more rapid increase in IMT (13 μm/year, 95% CI,3,24). These findings suggest a role for FGF23 in HIV-positive populations in identifying patients at greater risk for cardiovascular and kidney disease.

## Introduction

Fibroblast growth factor-23 (FGF23) is a 32-kDa glycoprotein secreted by bone osteocytes and osteoblasts to circulate as an active full-length protein and shorter, inactive fragments [1,2]. It plays a central role in phosphate homeostasis by stimulating renal phosphate excretion via downregulation of sodium-dependent phosphate transporter 2a and 2c (NPT2a and NPT2c) in the renal proximal tubule [3,4]. FGF23-null mice display increased renal phosphate reabsorption with elevated serum phosphate concentrations [4]. FGF23-mediated signaling is dependent on transmembrane protein, Klotho, that functions as co-receptor for FGF23 [5].

Consistent with its role in bone mineral regulation, which becomes disordered in patients with kidney disease, several studies demonstrated that FGF23 levels rise as kidney function declines, even among individuals with early chronic kidney disease (CKD) [6–10]. Moreover, higher FGF23 levels are strongly associated with mortality in this population [11,12]. The increase in mortality risk was demonstrated to be largely driven by an association between elevated FGF23 concentrations and cardiovascular events, independent of glomerular filtration rate (GFR) and established cardiovascular risk factors [13–17].

HIV-positive individuals are at increased risk for both cardiovascular and kidney disease. Contributors to cardiovascular disease (CVD) may include CKD itself [18] in addition to traditional cardiovascular risk factors (e.g., age, sex, diabetes, hypertension, cigarette smoking, and hypercholesterolemia), direct or indirect effects of HIV infection (including inflammation and immune activation), or adverse effects of HIV therapy [19–22]. To date, there are few data available addressing the potential role of FGF23 in kidney and cardiovascular disease in HIV-positive individuals.

In this study, we predominantly focus on the associations of baseline FGF23 serum concentration and subsequent changes in markers of kidney and cardiovascular disease. our objectives are to 1) assess for systematic differences in FGF23 levels in HIV-positive and HIV-negative subjects without clinically decreased kidney function, 2) assess factors associated with FGF23 levels, and 3) evaluate associations of FGF23 levels with cross-sectional and longitudinal indicators of CKD and CVD in HIV-positive participants.

## Materials and Methods

### Study design and population

The Mr. Bean study is a cohort in Baltimore, MD to assess demographic, behavioral, and viral (HIV and hepatitis C virus [HCV]) factors in the progression of kidney and cardiovascular disease [23]. We recruited HIV-positive participants from the Johns Hopkins HIV clinic and HIV-negative participants from the community in a 2:1 ratio. We recruited HIV-negative subjects through local media advertising and from a cohort of persons with a history of injection drug use, the latter to achieve comparable behavioral characteristics and HCV seropositivity rates to the HIV-positive sample. Inclusion criteria included age 18 years or older and estimated glomerular filtration rate (GFR) ≥ 60 mL/min/1.73 m^2^ (by MDRD equation [24]), the latter because a primary objective of the study was to track measured GFR changes over time in subjects with initially normal estimated kidney function. Exclusion criteria included history of radiocontrast allergy, pregnancy, diabetes mellitus, uncontrolled hypertension (systolic blood pressure > 160 mm Hg or diastolic blood pressure > 100 mm Hg), collagen vascular disease, or life threatening comorbidity. We confirmed HIV and HCV status through medical records and serologic testing. After screening and enrollment, participants completed a baseline study visit and up to three annual follow-up visits.

### Data collection and measurements

A summary of the data collection protocol for the Mr. Bean study is outlined in Table 1. At each study visit, we collected demographic, behavioral, clinical, and pharmacologic data by interview and medical record review, and measured blood pressure, height, and weight. Laboratory testing included plasma concentrations of creatinine, phosphorous, parathyroid hormone (PTH), high-sensitivity C-reactive protein, plasma cholesterol, and cholesterol subfractions. Activated CD8+ lymphocytes were measured by flow cytometry as the percentage of CD8+ cells expressing CD38+ and HLA-DR surface markers. We measured intact FGF23 concentrations in duplicate from baseline plasma samples (Immutopics, Inc., San Clemente, CA), inter-assay coefficient of variation 1.74%. We measured urine concentrations of creatinine, albumin, protein, and phosphorus from random urine samples.

**Table 1:** Data Collection Protocol for the Mr. Bean Study.

Assessment	Study visit (months)						
	0	6	12	18	24	30	36
Interview							
Demographics	●						
Behavioral survey	●	●	●	●	●	●	●
Medication review	●	●	●	●	●	●	●
Anthropomorphic							
Height, weight	●		●		●		●
Blood pressure	●		●		●		●
Blood tests							
Creatinine	●		●		●		●
Lipid panel	●		●		●		●
Glycosylated hemoglobin	●		●		●		●
High-sensitivity CRP	●		●		●		●
Activated CD8 lymphocytes	●		●		●		●
Phosphorus	●		●		●		●
HIV RNA^1^	●		●		●		●
CD4 cell count^1^	●		●		●		●
Parathyroid hormone	●						
HCV antibody	●						
FGF23	●						
Urine tests							
Creatinine	●	●	●	●	●	●	●
Albumin	●	●	●	●	●	●	●
Protein	●	●	●	●	●	●	●
Dipstick	●		●		●		●
Phosphorus	●		●		●		●
Urine drug screen	●	●	●	●	●	●	●
Iohexol GFR	●		●		●		●
Pulse wave velocity	●		●		●		●
Carotid ultrasound and IMT	●				●		

GFR, glomerular filtration rate; IMT, intima-media thickness

^1^ Testing done in HIV-positive participants

We measured GFR directly at each study visit using iohexol disappearance from plasma. A research nurse placed two peripheral intravenous catheters and infused a weighted dose of iohexol (5 mL, GE Healthcare, Amersham Division, Princeton, NJ) into one catheter and drew blood samples from the second catheter at 10, 30, 120, and 240 minutes following the infusion. The Schwartz Laboratory at the University of Rochester subsequently measured iohexol plasma concentrations, using high performance liquid chromatograph, and calculated measured GFR (mGFR) using a two-compartment model, described previously [25].

Trained sonographers used a Toshiba Aplio Ultrasound System, with a linear array transducer (PL-704) that has a frequency range from 4.8–11.0 MHz, to obtain B-mode ultrasound images of the common and internal carotid arteries bilaterally at baseline and at 24-month follow-up. Technicians at the Atherosclerosis Imaging Research Program at the University of Wisconsin, who were masked to participant characteristics, used Arterial Health Package software (Siemens Medical, Malvern, PA) for semi-automated IMT measurement and plaque scoring [26]. The presence of plaque, which was defined as a focal area of intima-media thickening ≥ 1.5 mm or 50% thicker than the neighboring wall, was assessed in 12 anatomical segments in the common and internal carotid arteries bilaterally. Carotid intima-media thickness (IMT) was measured in the right common and right internal carotid arteries. Carotid IMT measurements were performed in triplicate and mean-mean measures for common and internal carotid arteries were used in analyses.

Trained technicians measured pulse wave velocity (PWV) annually by applanation tonography [27,28]. Technicians measured PWV with participants in supine position and electrocardiography electrodes placed on each forearm and on the left calf. Measurements of height, weight, brachial blood pressure, distance from the center of the suprasternal notch to the carotid pulse site, and distance from the suprasternal notch to the femoral pulse site were entered into the SphygmoCor CvMS software (West Ryde, Australia). Waveform at the carotid and femoral pulse sites were captured with the tonometer, with PWV calculated by the software.

### Definitions and statistical analysis

Urine albumin-creatinine ratio (ACR) was measured at baseline and every 6 months thereafter. Consistent with guidelines [29], we defined albuminuria as an ACR ≥ 30 mg/g. Progressive albuminuria was defined as a follow-up ACR that was ≥2-fold higher than baseline and ≥30 mg/g. Fractional excretion of phosphorous (FE_phos_) was calculated as [(urine phosphorous concentration) *(serum creatinine concentration)/ (urine creatinine concentration) *(serum phosphorous concentration)] *100. Carotid plaque progression was defined as carotid plaque identified in more anatomical segments at 24 months than at baseline.

We compared demographic, behavioral, and laboratory characteristics in HIV-negative and HIV-positive subjects, using Fisher’s exact test and Wilcoxon rank-sum tests for categorical and continuous variables, respectively. We assessed factors associated with baseline FGF23 plasma concentration using log-transformed FGF23 values in linear regression models with exponentiated coefficients, which can be interpreted as the ratio of geometric means. We stratified univariate models by HIV status and assessed for statistical interactions. In the HIV-positive group, we constructed a multivariate model that initially included all variables that were statistically significantly associated (P<0.05) with FGF23 in univariate analyses, and used backward stepwise selection to select covariates for the final model (P≤0.1 to remain in model). We used linear regression to assess the cross-sectional association of log-transformed FGF23 at baseline with mGFR, common and internal carotid IMT, and PWV, and logistic regression to assess the association of FGF23 with carotid plaque (any) and albuminuria. Finally, we assessed the association of baseline FGF23 with longitudinal changes in markers of CKD and CVD, using mixed linear models to assess mGFR and PWV trajectory, linear regression to assess annualized change in right common and internal carotid IMT, and logistic regression to assess progressive albuminuria and carotid plaque progression.

### Ethics statement

Participants provided written informed consent and the Johns Hopkins Medicine Institutional Review Board approved the study.

## Results

### Cohort Characteristics

Among the 290 participants with a baseline measurement, 190 participants were HIV-positive. The median (25^th^ percentile [P_25_], 75^th^ percentile [P_75_]) FGF23 plasma concentrations were similar in HIV-negative (8.9 pg/mL, 6.5, 13.8) and HIV-positive participants (8.4 pg/mL, 6.5, 13.2) (P = 0.53). Overall, the cohort was 29% female, 93% black, had a median age of 49 years, and 46% were infected with hepatitis C virus (HCV) (Table 2). Compared with HIV-negative participants, HIV-positive participants were significantly more likely to be female, hypertensive, infected with HCV, current cocaine users, have albuminuria, and to have lower baseline GFR as measured by plasma iohexol clearance (mGFR). As anticipated, HIV-positive subjects also had significantly higher percentages of activated CD8 lymphocytes. HIV-positive participants were also more likely to be receiving vitamin D supplementation, antihypertensive medication, and HMG CoA reductase inhibitors.

**Table 2:** Baseline characteristics of the cohort overall, and stratified by HIV status.

	Cohort overall (n = 290)	HIV-negative participants (n = 100)	HIV-positive participants (n = 190)	P value^a^
Hypertension, n (%)	87 (30)	21 (21)	66 (35)	0.016
Body mass index, kg/m2, median (P_25_, P_75_)	26.0 (22.9, 31.4)	26.5 (23.5, 32.6)	25.5 (22.8, 30.9)	0.15
Systolic blood pressure, mm Hg, median (P_25_, P_75_)	122 (111, 132)	126 (113, 135)	119 (111, 132)	0.007
Current smoker, n (%)	181 (62)	60 (60)	121 (64)	0.61
Risky alcohol use, n (%)	81 (28)	31 (31)	50 (26)	0.41
Current cocaine use, n (%)	105 (36)	28 (28)	77 (41)	0.040
Serum creatinine, mg/dL, median (P_25_, P_75_)	0.9 (0.8, 1.1)	1 (0.8, 1.1)	0.9 (0.8, 1.1)	0.18
Iohexol-based GFR, mL/min/1.73m^2^	103 (88, 119)	108 (95, 125)	101 (86, 116)	0.01
Albumin-creatinine ratio ≥ 30 mg/g, n (%)	41 (14)	8 (8)	33 (17)	0.033
FGF23 level, pg/ml, median (P_25_, P_75_)	8.46 (6.48, 13.39)	8.89 (6.45, 13.83)	8.35 (6.52, 13.19)	0.53
Serum phosphorus, mg/dL, median (P_25_, P_75_)	3.5 (3.2, 3.9)	3.4 (3.0, 3.9)	3.5 (3.2, 3.9)	0.075
Fractional excretion of phosphorus, %, median (P_25_, P_75_)	10.3 (7.0, 13.8)	9.8 (6.7, 13.2)	10.5 (7.2, 14.1)	0.21
Parathyroid hormone, pg/mL, median (P_25_, P_75_)	43 (34, 56)	45 (34, 60)	42 (33, 54)	0.17
Glycosylated hemoglobin, %, median (P_25_, P_75_)	5.5 (5.2, 5.7)	5.5 (5.3, 5.8)	5.4 (5.1, 5.7)	0.024
Ratio of total to HDL cholesterol, median (P_25_, P_75_)	3.1 (2.5, 4.2)	3.1 (2.4, 4.1)	3.1 (2.5, 4.2)	0.87
High sensitivity CRP, mg/L, median (P_25_, P_75_)	1.8 (0.6, 4.5)	1.9 (0.8, 5.5)	1.7 (0.6, 4.2)	0.34
Activated CD8 lymphocytes^b^, %, median (P_25_, P_75_)	23 (12, 38)	11 (8, 20)	31 (19, 48)	<0.001
Vitamin D supplement, n (%)	22 (8)	3 (3)	19 (11)	0.035
Non-steroidal anti-inflammatory drug use, n (%)	178 (61)	59 (59)	119 (63)	0.61
Antihypertensive medication, n (%)	81 (28)	19 (19)	62 (33)	0.014
ACE inhibitor or ARB, n (%)	38 (13)	8 (8)	30 (16)	0.068
HMG CoA reductase inhibitor, n (%)	31 (11)	4 (4)	27 (14)	0.008
History of CDC category C opportunistic condition (%)	-	-	48 (25)	-
Current CD4 count, cells/mm^3^	-	-	467 (248, 627)	-
HIV RNA <400 copies/mL, n (%)	-	-	152 (79)	-

P25 and P75, 25^th^ and 75^th^ percentiles, respectively; GFR, glomerular filtration rate; HDL, high-density lipoprotein; CRP, C-reactive peptide; ACE, angiotensin converting enzyme; ARB, angiotensin II receptor blocker; HMG CoA, 3-hydroxy-3-methylglutaryl-coenzyme A; CDC, Centers for Disease Control and Prevention.

^a^ Fischer’s exact test and Wilcoxon rank-sum test for categorical and continuous variables, respectively.

^b^ Percentage of CD8 lymphocytes expressing CD38 and HLA-DR surface markers.

### FGF23 plasma concentrations and baseline factors in HIV-negative and HIV-positive participants

As displayed in Table 3, among HIV-positive subjects, factors associated with higher FGF23 levels at baseline included being female (adjusted ratio of geometric means; 95% confidence interval [CI]), 1.46;1.21, 1.76), serum phosphorus (1.21 per mg/dL higher; 1.03, 1.42), and HCV coinfection (1.31;1.10, 1.56). Parameters of uncontrolled HIV infection including lower CD4 count, activated CD8+, and non-suppressed HIV RNA were all significantly associated with FGF23 level in the unadjusted model. In the adjusted model, non-suppressed viral load remained significantly associated with higher FGF23 levels (adjusted ratio of geometric means, 1.27, 95% CI 1.01, 1.60). No association was observed between baseline FGF23 and parathyroid hormone level, FE_phos_, use of vitamin D supplementation, or treatment with tenofovir or abacavir.

**Table 3:** Associations of baseline factors with fibroblast growth factor-23 plasma concentrations in HIV-negative and HIV-positive participants.

Factor	HIV-negative subjects		HIV-positive subjects	
	Unadjusted ratio of geometric means	P for interaction between HIV-positive and HIV-negative groups	Unadjusted ratio of geometric means	Adjusted^a^ ratio of geometric means
Female	0.98 (0.73, 1.34)	0.008	**1.63 (1.34, 1.98)**	**1.46 (1.21, 1.76)**
Non-African American	0.95 (0.61, 1.48)	NS	0.97 (0.64, 1.48)	-
Age, per 10 years higher	0.99 (0.84, 1.15)	NS	0.94 (0.83, 1.07)	-
Hepatitis C infection	0.87 (0.67, 1.12)	0.012	**1.33 (1.10, 1.62)**	**1.31 (1.10, 1.56)**
Hypertension	0.91 (0.68, 1.22)	NS	1.21 (0.98, 1.48)	-
Body mass index, per 1 kg/m^2^ higher	1.01 (0.99, 1.03)	NS	1.00 (0.99, 1.02)	-
Systolic blood pressure, per 10 mm Hg higher	1.00 (0.92, 1.09)	NS	1.04 (0.98, 1.10)	-
Current smoker	0.86 (0.67, 1.10)	NS	1.05 (0.86, 1.29)	-
Risky alcohol use^b^	0.97 (0.75, 1.25)	NS	1.04 (0.83, 1.30)	-
Current cocaine use	0.97 (0.74, 1.27)	NS	0.93 (0.76, 1.14)	-
Iohexol GFR, per 10 mL/min/1.73m^2^ higher	0.97 (0.92, 1.03)	NS	0.97 (0.92, 1.01)	-
Albumin-creatinine ratio ≥ 30 mg/g	1.00 (0.64, 1.56)	NS	**1.49 (1.15, 1.92)**	**1.28 (1.01, 1.62)**
Serum phosphorus, per 1 mg/dL higher	1.23 (0.99, 1.53)	NS	**1.32 (1.12, 1.56)**	**1.20 (1.03, 1.40)**
Fractional excretion of phosphorus, per 5% higher	1.02 (0.89, 1.15)	NS	1.05 (0.96, 1.15)	-
Parathyroid hormone, per 10 pg/mL higher	1.02 (0.96, 1.08)	NS	1.02 (0.96, 1.07)	-
Glycosylated hemoglobin, per 1% higher	0.93 (0.71, 1.22)	NS	0.95 (0.75, 1.20)	-
Total to HDL cholesterol ratio, per 1 unit higher	1.04 (0.94, 1.15)	NS	1.00 (0.92, 1.08)	-
High sensitivity CRP (units), per doubling	1.05 (0.98, 1.11)	NS	0.98 (0.93, 1.03)	-
Activated CD8 lymphocytes^c^, per 10% higher	**0.88 (0.79, 0.98)**	0.001	**1.09 (1.03, 1.15)**	-^d^
Vitamin D supplement	1.14 (0.60, 2.18)	NS	0.76 (0.54, 1.06)	-
Nonsteroidal anti-inflammatory drug	1.20 (0.94, 1.53)	NS	1.02 (0.83, 1.25)	-
Antihypertensive medication	0.92 (0.68, 1.25)	NS	1.19 (0.97, 1.46)	-
ACE inhibitor or ARB	0.90 (0.58, 1.40)	NS	1.09 (0.83, 1.42)	
HMG CoA reductase inhibitor	0.91 (0.49, 1.68)	NS	**0.74 (0.56, 0.98)**	-^d^
History of CDC category C opportunistic condition	-	-	1.15 (0.92, 1.44)	-
Nadir CD4 count, per 50 cells/mm^3^ higher	-	-	0.98 (0.96, 1.01)	-
HIV RNA > 400 copies/mL	-	-	**1.49 (1.18, 1.88)**	**1.27 (1.01, 1.60)**
Current CD4 count < 350 cells/mm^3^	-	-	**1.35 (1.10, 1.64)**	1.21 (0.99, 1.46)
Abacavir use	-	-	1.01 (0.77, 1.32)	-
Tenofovir use	-	-	1.11 (0.90, 1.38)	-
Ritonavir-boosted protease inhibitor use	-	-	1.12 (0.91, 1.38)	-

Estimates shown in bold are statistically significant (P<0.05)

NS, non-significant; GFR, glomerular filtration rate; HDL, high-density lipoprotein; CRP, C-reactive peptide; HMG CoA, 3-hydroxy-3-methylglutaryl-coenzyme A.

^a^ Adjusted for factors shown in column.

^b^ Risky alcohol use defined by a score > 3 in men or >2 women on the Alcohol Use Disorders Identification Test-C instrument.

^c^ Percentage of CD8+ lymphocytes expressing CD38+ and HLA-DR surface markers.

^d^ Factor removed from multivariate model by backwards selection (P>0.1)

Among the HIV-negative participants, the only factor that was statistically significantly associated with FGF23 levels was the percentage of activated CD8+ cells. Interestingly, a higher percentage of CD8+ cells was associated with a significantly lower FGF23 levels in HIV-negative individuals, whereas the opposite was observed among HIV-positive individuals (P = 0.001 for interaction). Additionally, there were two other variables that had evidence of statistically significant interaction according to HIV status. Female sex and HCV infection had weak associations with FGF23 levels in HIV-negative participants, but were strongly associated with higher FGF23 levels in HIV-positive participants. As shown in Table 2, only 13% of the entire cohort were treated with angiotensin converting enzyme inhibitors (ACEI) or angiotensin receptor blockers (ARB), but there was no observed association between FGF23 level and ACEI or ARB use or interaction by HIV status (Table 3).

In supplementary analyses to explore sex differences in FGF23 levels, we found that plasma phosphorus concentrations were significantly higher in women compared with men (median 3.6 vs. 3.5 mg/dL, P = 0.05) as were plasma parathyroid hormone concentrations (median 49 vs 42 pg/mL, P = 0.009). However, there were no statistically significant difference by sex in FE_phos_, or use of tenofovir, abacavir, or HMG CoA reductase inhibitors. Additionally, we found no evidence that FGF23 levels were associated with age among women (data not shown).

### FGF23 plasma concentration and cross-sectional and longitudinal indicators of cardiovascular disease and kidney

**Fig 1:**
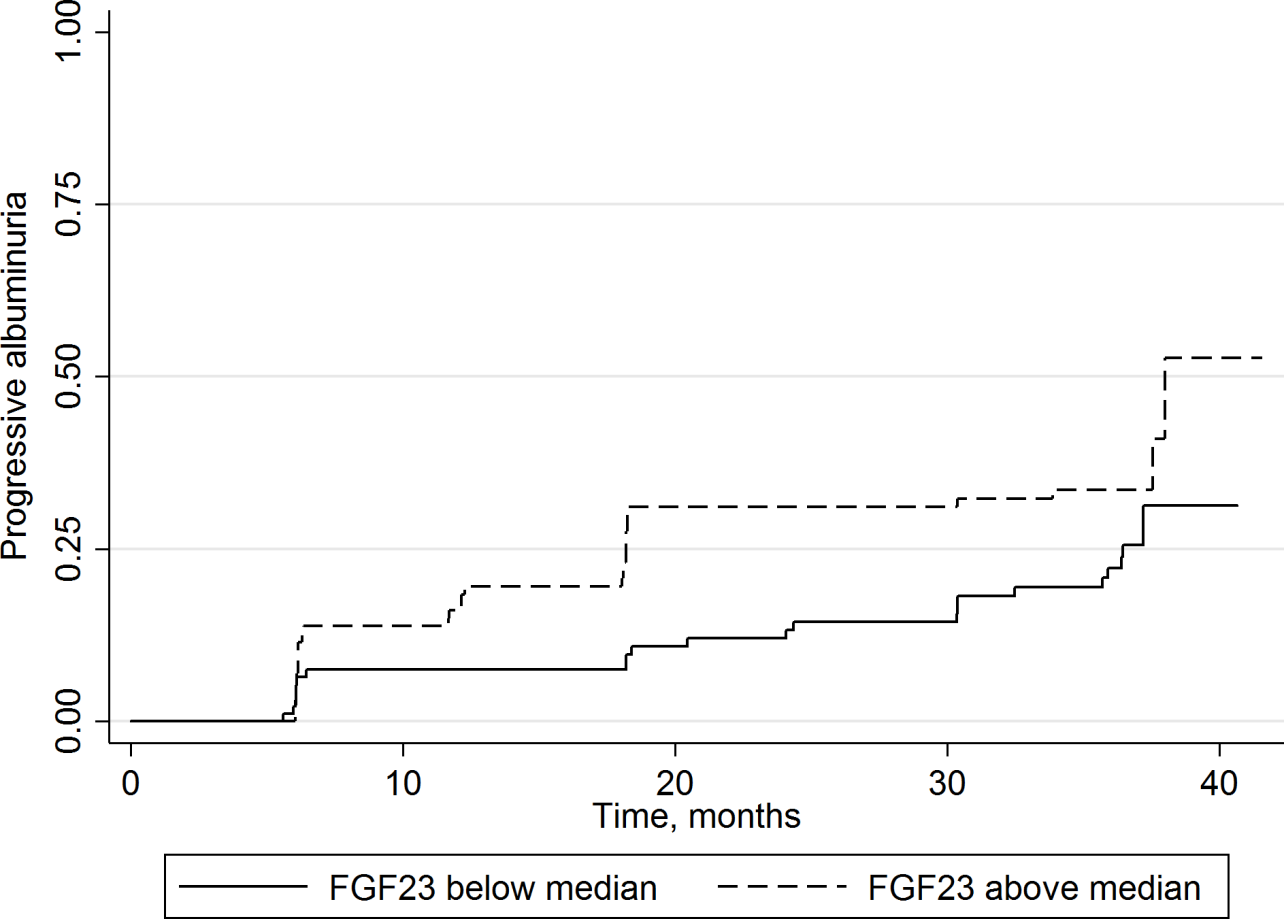
Time to progression to albuminuria by FGF23 level. Kaplan-Meier plot showing time to progressive albuminuria according to baseline FGF23 plasma concentration below or above the median (8.4 pg/mL) among HIV-positive individuals (P = 0.05, logrank test).

In cross-sectional analyses of baseline markers of kidney and CVD, higher FGF23 levels were significantly associated with higher pulse wave velocity and albuminuria in univariate analyses (Table 4). However, when adjusted for demographic and CVD risk factors (including smoking, hypertension, and hyperlipidemia) these associations were no longer statistically significant. Conversely, in the longitudinal analysis and adjusted for the same factors, higher baseline FGF23 was significantly and independently associated with a more rapid increase in internal carotid IMT (13 μm/year, 95% CI, 3, 24) and risk of progressive albuminuria (odds ratio 1.48 [95% CI]: 1.05, 2.08). Fig 1. displays the Kaplan-Meier plot for FGF23, stratified at the median level, and time to progressive albuminuria. These associations were not modified by ACEI or ARB use (data not shown).

**Table 4:** Associations of a 2-fold increase in fibroblast growth factor-23 plasma concentration at baseline with cross-sectional and longitudinal indicators of kidney and cardiovascular disease

Kidney and cardiovascular disease markers	Estimate units	Unadjusted estimate (95% CI)	Adjusted^a^ estimates (95% CI)
**Baseline measures (cross-sectional associations)**			
Continuous measures (linear regression)			
mGFR	mL/min/1.73m^2^	-2.31 (-5.42, 0.81)	-1.69 (-4.89, 1.51)
Common carotid IMT	μm	3 (-30, 35)	11 (-20, 44)
Internal carotid IMT	μm	-29 (-83, 25)	-38 (-96, 20)
Pulse wave velocity	m/s	**0.39 (0.10, 0.68)**	0.27 (-0.02, 0.56)
Binary measures (logistic regression)			
Albumin-creatinine ratio ≥ 30 mg/g	Odds ratio	**1.65 (1.16, 2.33)**	1.45 (0.98, 2.15)
Presence of carotid plaque^b^	Odds ratio	1.06 (0.78, 1.42)	1.05 (0.73, 1.51)
**Longitudinal measures (change after baseline)**			
Continuous measures (linear models)			
mGFR slope	mL/min/1.73m^2^ per year	-0.58 (-1.57, 0.40)	-0.84 (-1.88, 0.20)
Common carotid IMT	μm per year	3 (-4, 10)	3 (-5, 10)
Internal carotid IMT	μm per year	10 (0, 21)	**13 (3, 24)**
Pulse wave velocity	m/s per year	-0.07 (-0.17, 0.04)	-0.05 (-0.17, 0.06)
Binary measures (logistic regression)			
Progressive albuminuria^c^	Odds ratio	**1.44 (1.05, 1.96)**	**1.48 (1.05, 2.08)**
Carotid plaque progression^d^	Odds ratio	0.94 (0.67, 1.32)	0.85 (0.59, 1.24)

Estimates shown in bold are statistically significant (P<0.05)

CI, confidence interval; mGFR, glomerular filtration rate measured by iohexol disappearance from plasma; IMT, intima-media thickness.

^a^ Estimates adjusted for sex, race, age, smoking, hypertension diagnosis, systolic blood pressure, and total cholesterol/high-density lipoprotein cholesterol ratio.

^b^ Carotid plaque defined as a focal area of intima-media thickening ≥ 1.5 mm or 50% thicker than the neighboring wall.

^c^ Progressive albuminuria defined as an albumin-creatinine ratio during follow-up that is at least 2-fold higher than the baseline values and ≥ 30 mg/g

^d^ Carotid plaque progression defined as detection of plaque in at least one new segment compared to baseline.

## Discussion

In this longitudinal study of non-diabetic individuals with clinically normal kidney function at baseline, we found no difference in baseline FGF23 levels in HIV-positive and demographically and clinically similar HIV-negative participants. We found that that among HIV-positive participants that higher FGF23 levels at baseline were associated with more rapid carotid IMT progression and with progressive albuminuria. We also found among HIV-positive participants that several factors were independently associated with higher FGF23 levels at baseline including female sex, serum phosphorus, HCV coinfection, and non-suppressed HIV RNA. In contrast, we found no association between FGF23 level at baseline with mGFR or with the use of tenofovir, abacavir, boosted protease inhibitor, or vitamin D.

We found a strong independent assciation between higher FGF23 levels at baseline and progressive albuminuria over 36-month follow-up. Our findings support those reported in the Heart and Soul Study, a general population cohort of adults with stable CVD, in which FGF23 levels were linearly associated with levels of albuminuria [30]. These findings suggest that FGF23 may play a potential role in identifying patients at high risk of CKD in HIV-positive individuals, independent of traditional risk factors. These data also suggest that FGF23 is an early indicator of kidney injury in this population.

The association of higher FGF23 level at baseline with more rapid carotid IMT in HIV-positive participants is consistent with its associated role in CVD in both general population and in patients with CKD [9,14,15,31]. It is also in line with an earlier finding that higher circulating FGF23 is independently associated with a 10-year cardiovascular risk score >20% in 51 HIV positive cohort[32]. Another recent small study suggested that FGF23 levels in HIV-positive individuals were significantly associated with metabolic disturbances and fat distribution, but not with Framingham cardiovascular risk score [33].

Viral effects of HIV and HCV on bone, where FGF23 is produced, are difficult to ascertain because of the multitude of confounding factors. HIV-positive men and women had a 3.7-fold increased risk of osteoporosis (T-score≤−2.5) and 6.4-fold increased risk of low bone mineral density (T-score ≤1.0) compared with HIV-negative persons [34]. HCV coinfection and drug use have also been associated with lower bone mineral density in HIV-positive populations [35]. We found significant independent associations of FGF23 level with both non-suppressed HIV RNA and with HCV coinfection. The mechanisms underlying these observations remains speculative. HIV or HCV infection may trigger the release of FGF23 with subsequent bone phosphate loss leading to bone demineralization in this population. Alternatively, FGF23 may be marker of an inflammatory response to these viral infections. Interestingly, we found that the associations of FGF23 with activated CD8+ cells and with HCV infection were modified by HIV status. The median percentage of activated CD8+ cells was nearly 3-fold higher in HIV-positive than HIV-negative individuals in our study. Among HIV-positive individuals, activated CD8+ cells are strongly correlated with viral load, partially (but not completely) normalize with durable viral suppression on antiretroviral therapy, and strongly predict HIV-disease progression and all-cause mortality [23,36,37]. Our data suggest that FGF23 has no association with lower levels of activated CD8+ cells seen in HIV-negative persons, but has a positive correlation with activated CD8+ cells at the dramatically elevated levels seen in HIV-positive persons. Similarly, our data suggest that HCV infection in HIV-negative persons is insufficient to affect FG23 levels, while HCV co-infection among HIV-positive individuals is associated with increased FGF23 levels.

Our study is limited by the lack of bone mineral density measures although qualification of any of these hypotheses may in fact require bone biopsy given the limitations of existing imaging studies in separating the effect of all of these various factors on bone. Finally, compared to other ART, tenofovir is associated with greater decrease in bone density and renal phosphaturia [38,39]. The lack of association of FGF23 with tenofovir in our cohort is consistent with a prior study demonstrating that the phosphaturic effect of tenofovir is independent of FGF23 level [40] but is in contrast to one case report suggesting otherwise [41].

The independent association of FGF23 with female gender and serum phosphorus level is in line with previous studies. Ix *et al*. demonstrated higher FGF23 in older women compared with similarly aged men and that higher FGF23 consistently tracked with higher serum phosphorus levels [42]. Estradiol is phosphaturic via an inhibitory effect on the NaPi-IIa cotransporter in renal proximal tubules. Consequently, the decrease in estradiol production in menopause leads to decreased phosphate excretion in the urine and increased phosphorus levels in the blood. The observed hyperphosphatemia in post-menopausal women presumably leads to increased production of FGF23 by bone and this has been demonstrated both experimentally and clinically [43–47]. In HIV-positive participants, we found that that the sex association with FGF23 levels was attenuated when adjusted for serum phosphorus and other factors, but that it retained statistical significance, suggesting that serum phosphorus does not completely account for sex differences in FGF23. Although information on menopausal status was lacking in participant women, these data are suggestive of a potential FGF23 role in the acceleration in CVD risk that occurs after menopause in women [48]. The basis for the modifying effect of HIV status on the sex association with FGF23 is not clear, and should be interpreted with caution given the small number women in the HIV-negative group.

Although our study is limited by its relatively small sample size, measurements of FGF23 at only one-time point, and the lack of vitamin D measurements, and DEXA scan, it provides the first evaluation of its potential role in cardiovascular and kidney disease in non-diabetic HIV-positive participants with clinically normal kidney function at baseline compared and with longitudinal follow-up for 3 years. We acknowledge that our sample was predominantly African American and that our results may not extrapolate to non-African American racial groups.

In conclusion, we found no differences in FGF23 levels in middle-aged, predominantly African American HIV-positive participants and a demographically and clinically comparable group of HIV-negative individuals. However, among HIV-positive individuals, we found significant associations of HCV co-infection and unsuppressed HIV RNA levels with higher FGF23 levels that require further exploration of the interaction between viral infection and/or immune activation and bone disease. Furthermore, our findings of the independent associations of higher FGF23 levels at baseline with more rapid carotid IMT progression and with progressive albuminuria suggest a role for FGF23 in cardiovascular and kidney disease in HIV-positive populations.
